# *Chlorella vulgaris* functional alcoholic beverage: Effect on propagation of cortical spreading depression and functional properties

**DOI:** 10.1371/journal.pone.0255996

**Published:** 2021-08-09

**Authors:** Danielli M. M. Dantas, Thiago B. Cahú, Carlos Yure B. Oliveira, Ricardo Abadie-Guedes, Nathalia A. Roberto, Werlayne M. Santana, Alfredo O. Gálvez, Rubem C. A. Guedes, Ranilson S. Bezerra

**Affiliations:** 1 Departamento de Bioquímica, Universidade Federal de Pernambuco (UFPE), Recife, Brazil; 2 Departamento de Pesca e Aquicultura, Universidade Federal Rural de Pernambuco (UFRPE), Recife, Brazil; 3 Departamento de Nutrição, Universidade Federal de Pernambuco (UFPE), Recife, Brazil; Institute for Biological Research, University of Belgrade, SERBIA

## Abstract

Recent advances in microalgae biotechnology have proven that these microorganisms contain a number of bioactive molecules, that can be used as food additives that help prevent disease. The green microalga *Chlorella vulgaris* presents several biomolecules, such as lutein and astaxanthin, with antioxidant capacity, which can play a protective role in tissues. In this study, we produced and analyzed a *C*. *vulgaris* functional alcoholic beverage (produced using a traditional Brazilian alcoholic beverage, *cachaça*, and *C*. *vulgaris* biomass). Assays were conducted *in vitro* by radical scavenging tests, and *in vivo*, by modeling cortical spreading depression in rat brains. Scavenging radical assays showed that consumption of the *C*. *vulgaris* alcoholic beverage had a DPPH inhibition of 77.2%. This functional alcoholic beverage at a concentration of 12.5 g L^-1^ significantly improved cortical spreading depression velocity in the rat brains (2.89 mm min^-1^), when compared with *cachaça* alone (3.68 mm min^-1^) and control (distilled water; 3.25 mm min^-1^). Moreover, animals that consumed the functional beverage gained less weight than those that consumed just alcohol and the control groups. These findings suggest that the *C*. *vulgaris* functional alcoholic beverage plays a protective physiologic role in protecting brain cells from the effects of drinking ethanol.

## Introduction

Ethyl alcohol consumption is one of the most common causes of general health disabilities, lower life expectancy, and also is associated to many chronic diseases [1, 2]. Due to the neurotoxic effect of alcohols, excessive ethyl alcohol consumption has been linked to an increased risk of some diseases, such as dementia, [3]. Controversially, epidemiological studies have indicated that moderate consumption of red wine may have a positive impact on human health [4, 5]. Phytochemicals, chemical compounds produced by plants, may be found in various beverages, such as wine. People who are moderate wine drinkers (three to four glasses a day) have a lower incidence of dementia compared to nondrinkers [6–9]. The possible mechanisms for these protective effects include antioxidant and anti-inflammatory properties as well as higher plasma apolipoprotein E levels [8, 9]. Therefore, moderate wine drinking is seen as a possible preventive measure against senile dementia, even though a direct demonstration of the protective effects of wine or a delineation of its mechanisms have not yet been fully elucidated [7].

The interest in biocompounds with antioxidant and antibacterial properties, such as polyphenols and carotenoids, has been increasing over the years. Due to the diversity of these compounds, they can exhibit a broad range of biological activities. For example, some plants and algae are particularly rich in polyphenols identified as having potential antioxidant activity [10], while other phenolic compounds and carotenoids have been most commonly related to free radical scavenging activity [11]. Therefore, the study of such biological compounds has been identified as a promising research field.

A growing attention to functional food has driven research into the physiological effects of high biological value components [12]. Microalgae are among the most promising sources of bioactive compounds for new food products, which can be used to enhance the nutritional value of foods due to their well-balanced chemical composition and their potential anticancer, anti-diabetes, anti-inflammatory and antioxidant properties [13, 14]. The addition of microalgae biomass to food products has become an interesting method for providing nutritional supplementation with biologically active compounds, like fatty acids, essential amino acids, polysaccharides, vitamins, carotenoids, and others [15–17]. In this context, *Chlorella* and *Arthrospira* spp. (Spirulina) are already being commercialized in various forms, mainly as powders, tablets and capsules, that can be added to drinks or food. *Chlorella* spp., particularly *C*. *vulgaris*, have a wide variety of compounds including polysaccharides [18, 19], flavonoids [20] and polyphenols [21], with reported biological activities.

The concern over the nutritional value and beneficial effects of food and beverages, aiming a longer and healthier life expectancy, and the growing of vegan food market have a major impact on consumer acceptance of using algae as food source [22, 23]. The market of microalgae-based functional foods is very promising and grows with the demand of the population that seeks balanced foods that can improve physical and mental well-being of the consumers. In recent years, microalgae are being considered a potential functional food source to prevent, treat or ameliorate of various diseases such as cardiovascular diseases, cancer, inflammations, neurological disorders etc. [23–25].

The cortical spreading depression (CSD) phenomenon is an interesting electrophysiological model that is valuable for understanding the relationship between oxidative stress, nutrition, and neural development and function [26]. CSD has been studied rigorously in rodents and humans because of its clinical significance in neurologic disorders, which has contributed to better knowledge and clinical improvement in neural diseases. To gain a better understanding of the action of compounds from microalgae-based alcoholic extracts and their application to human health, particularly on the nervous system, we studied the effects of consumption of a *Chlorella* functional alcoholic beverage on changes in propagation of CSD in the cerebral cortex of young-adult rats treated chronically.

## Material and methods

### Microalgae production

The green microalga *Chlorella vulgaris* used in this study were obtained from the Laboratório de Produção de Alimento Vivo at the Universidade Federal Rural de Pernambuco, Brazil. The microalga inoculum was cultivated in semi-continuous mode, in both laboratory and pilot-scale. Cultures of *C*. *vulgaris* were conducted using autoclaved freshwater enriched with Provasoli medium in 2–L Erlenmeyer type glass flasks, under 150 μmol photons m^−2^ s^−1^, homogenized continuously with atmospheric air at 24±0.5°C. After increasing the cultivation to 100–L cultures, a 500-L pilot-scale fiberglass tank was used as the final stage of cultivation, using N:P:K (2:1:2) fertilizer as the culture medium. The pilot-scale tank was under ~1.850 μmol photons m^−2^ s^−1^ in a natural photoperiod (12:12h light/dark cycle). The microalgae cells were harvested at exponential growth phase by flocculation, using NaOH (1.0 M), and then freeze-dried (-50±1°C and 150×10^−3^ mbar) [15].

### Ultra-Performance Liquid Chromatography with coupled Mass Spectroscopy (UPLC–MS)

Chromatographic runs were performed in an ultra-performance liquid chromatography (UPLC, Acquity H–Class system, Waters Inc, Milford, MA, USA), with a C18 HSS 2.1 × 100 mm column and particle size of 1.8 μm (Waters Inc, Milford, MA, USA). The mobile phase was composed of 0.1% formic acid in acetonitrile (eluent A), 0.1% formic acid in ethyl acetate (eluent B) and 0.1% formic acid in methanol (eluent C), at an elution rate of 0.37 mL min^-1^ and an isocratic run, with a proportion of 10% A/40% B/50% C for 5 min, and sample injection volume of 10 μL. Column temperature was kept at 30°C, while the auto injector was at 10°C. The UPLC detection system was composed of a coupled mass spectrometer single quadrupole SQ Detector 2. Capilar voltage was 5.5 kV and cone voltage 50 V, the desolvation temperature was 350°C, and gas flow was 650 L h^-1^. Data acquisition was carried out in full scan mode, for masses between 100 and 1000 Da in positive ionization. Acquisition of chromatograms and mass spectra was performed with the software MassLynx™ (Waters Inc, Milford, MA, USA).

### Preparation of the *C*. *vulgaris* functional alcoholic beverage

Samples of dried microalgae biomass were extracted in a commercial Brazilian alcoholic beverage (Pitu Ltda., Recife, PE, Brazil) that contains 40% ethanol from sugar cane (*cachaça*), resulting in a hydroalcoholic microalgae extract (*C*. *vulgaris* functional alcoholic beverage, CFAB) at a final concentration of 12.5 g L^-1^. The intracellular compounds were extracted under 30 min of ultrasound disruption (40 kHz) in an ultrasonic batch (model Ultra Cleaner 1400, Ultrasonic Unique, Brazil) followed by chamber shaking for 2 h and centrifugation (2,486 g, Herolab UniCen MR Centrifuge, Wiesloch, BW, Germany) for 10 min to obtain the supernatant liquid. The CFAB was stored in a freezer (-20±1°C) and analyzed daily to evaluate the quality of the samples for antioxidant activity during the experiment. We established three days as the CFAB expiration date, after which a new microalgal hydroalcoholic extract was prepared.

### Antioxidant activity

The antioxidant activity of the *C*. *vulgaris* extracts was evaluated by 1, 1–diphenyl–2–picryl hydrazyl (DPPH) free-radical scavenging activity as reported by Dantas et al. [12]. The extracts were analyzed to evaluate the DPPH percentage of inhibition and a possible change over the days of the experiment.

### Experimental design

Wistar rats were reared in polypropylene cages (51 × 35.5 × 18.5 cm) in a room maintained at 23 ± 1°C with a photoperiod of 12 h (lights on 7:00 AM). A total of 27 rats were divided into three groups (at age of 55–73 days). For 18 days each group received by gavage (a method for administering substances directly into the stomach through a cannula inserted in the mouth) 4.5 mL kg^-1^ d^-1^ doses of *Chlorella* functional alcoholic beverage, CFAB (CFAB-group); *cachaça* (*cachaça*-group) and distilled water (control-group). All groups had free access to water and a diet of commercial laboratory feed (23% crude protein, Purina do Brazil Ltd, Paulínia, SP, Brazil).

### Electrophysiological recordings

Immediately after the gavage period, the CSD recording session was carried out, as previously described [27]. Briefly, under anesthesia with a mixture of 1 g kg^-1^ urethane plus 40 mg kg^-1^ chloralose (Sigma; injected intraperitoneally), a tracheal cannula was inserted and 3 trephine holes were made on the right side of the skull, aligned along the anteroposterior direction and parallel to the midline.

One hole was placed in the frontal bone (2 mm of diameter) and was used to apply the stimulus to elicit CSD. The other 2 holes were drilled in the parietal bone (3 to 4 mm in diameter) and were used to record the propagating CSD wave. The distance between the centers of contiguous holes was about 3 to 5 mm. Rectal temperature was continuously monitored and maintained at 37 ± 1° C with a heating blanket. At 20-min intervals, CSD was elicited by application, for 1 min, of a cotton ball (1 to 2 mm in diameter) soaked with 2% KCl solution (270 mM) placed on the frontal cortical surface through the hole drilled on that region. At the 2 parietal holes, both the slow change in direct current (DC) voltage and the reduction in the spontaneous cortical electrical activity accompanying CSD were continuously recorded for 4 h using a pair of Ag–AgCl Agar-Ringer electrodes (1 in each hole), as previously described [28]. A common reference electrode, of the same type, was placed on the nasal bones. The changes in DC voltage were recorded by connecting the electrodes to GRASS DC-amplifiers (Astro-Med Industrial Park, West Warwick, RI, USA), and the ECoG was recorded with AC-amplification (band pass filters set at 1 to 35 Hz range). Both DC-recording and ECoG were performed with a model 7–D GRASS chart recorder (Astro-Med Industrial Park, West Warwick, RI, USA). In some experiments, DC-recording was also computer-digitalized and recorded. The CSD propagation velocity was calculated from the time required for a CSD wave to cover the distance between the 2 cortical electrodes. In the measurement of CSD velocities, the initial point of each DC negative rising phase was used.

### Statistical analysis

A one-way analysis of variance (ANOVA), followed by the Tukey’s test, when necessary, was applied using a significance level of 0.05. Normality and homoscedasticity were evaluated by the Shapiro-Wilk and Levene tests, respectively [29].

### Ethics statement

The animals (male Wistar rats) were handled in accordance with the norms of the Ethics Committee for Animal Research of the Universidade Federal de Pernambuco, Brazil (where the experiments were conducted), which comply with the “Principles of Laboratory Animal Care” (National Institutes of Health, Bethesda, USA). The study was conducted after approval by the Ethics Committee on Animal Experimentation at UFPE (process number 23076.006249-2004-82). All electrophysiological recordings were performed under anesthesia, and subsequently, the rats were euthanized by an overdose of anesthetics. All efforts were made to minimize suffering.

## Results and discussion

**Fig 1:**
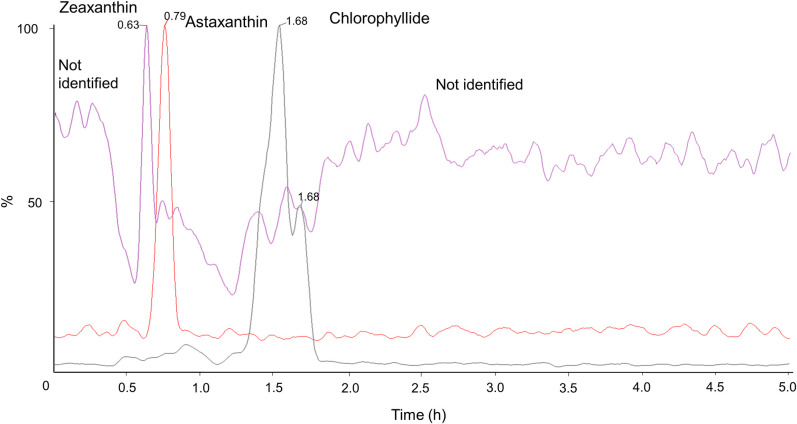
Chromatogram of the *Chlorella vulgaris* functional alcoholic beverage in ethyl acetate.

This study evaluated the production of an alcoholic beverage with the microalga *C*. *vulgaris* and both the antioxidant activity (*in vitro*) and the CSD model in brains of rats that consumed this functional beverage. Zeaxanthin, astaxanthin and chlorophyllide were the most abundant antioxidant pigments found in the CFAB (Fig 1). These four compounds contributed to almost 50% of the total antioxidant potential in the CFAB, with their isomers and derivatives comprising the rest. This information indicates that hydrophilic compounds, pigments and pheophytin were compounds with strong antioxidant activity and that can attach positive properties in CFAB [30]. Astaxanthin is the major carotenoid marketed in the world, and is already widely used for aquaculture purposes and also for human nutrition and supplementation [31], and a number of studies have demonstrated bioactive properties of this carotenoid [32, 33]. Furthermore, other carotenoids, such as lutein and violaxanthin, are commonly found in *C*. *vulgaris* biomass, but were not found in the present study. According to Yusof et al. [34], *C*. *vulgaris* has a higher concentration of carotenoids when grown under continuous lighting (i.e., 24:0 light/dark cycle). Thus, once cultures of *C*. *vulgaris* in the present study were conducted in natural photoperiod (i.e., 12:12 light/dark cycle) is likely that photoperiod had a negative influence on the carotenoid profile. In addition, other growing conditions can influence on the carotenoid profile in microalgae, such as nutritional metabolism, growth curve phase and salt stress [35, 36].

Growth performance of young-adult rats that received alcoholic beverages (CFAB or *cachaça*) and water are shown in Table 1. All animals survived and were in good health throughout the assays, however, the longevity of rats that did not consume alcoholic beverages was 9 days longer. In addition, rats that consumed alcoholic beverages had lower body weight and weight gain compared to the control group during the assays.

**Table 1:** Growth performance of young-adult rats (79–88 days of life) treated per gavage with *C*. *vulgaris* functional alcoholic beverage (CFAB), *cachaça* and water.

	Control	CFAB	*Cachaça*
**Final weight (g)**	361.8 ± 5.34^a^	292.2 ± 9.66^b^	297.8 ± 5.29^b^
**Weight gain (g)**	62.15 ± 18.74^a^	32.63 ± 9.84^b^	46.5 ± 14.02^ab^
**Age (days)**	88	79	79
**Radical scavenging (%)**	0	77.2	36.7

Data represent mean ± standard deviation of nine individuals for each treatment. Different letters on the same line indicate a significant difference by the Tukey’s *post-hoc* test (*p* <0.05).

These responses in weight gain are congruent with findings reported by Nuño et al. [37] who observed similar results when evaluating the effect of the diets containing microalgae *Isochrysis galbana* and *Nannochloropsis oculata* in nutritional treatment of diabetic rats. These authors reported that both healthy and diabetic rats that received a microalgae-based diet had a lower final weight than the control group (no microalgae), and suggested that consumption of the microalga *I*. *galbana* could be beneficial to rats with diabetes mellitus because it promoted body weight loss in healthy animals and helped to maintain weight in diabetic rats, while lowering glucose and cholesterol values. In addition, studies conducted with rats that received *Chlamydomonas reinhardtii* biomass showed that consuming lyophilized microalgae biomass improved body-weight loss and mitigated the extent of colonic damage [38, 39]. Thus, body-weight loss may be attributed to the anti-obesity effects of microalgae biomass intake, which facilitate a reduction in body fat and body-weight loss.

In a previous study, we observed the presence of bioactive compounds (phenols, flavonoids and tannins) in *C*. *vulgaris* extract. Furthermore, the extracts with solvents of ethanol and water showed DPPH percentage of inhibition of 37.2% and 68.5%, respectively, higher than the standardly used Gallic acid (28.7%), showing that they are potential inhibitors of cellular oxidation by free radicals [20]. Herein, CFAB and *cachaça* showed a percentage of inhibition of 77.2% and 36.7%, respectively. The level of DPPH inhibition of the extracts prepared did not change during the experiment, showing that the beverage’s functionalities were preserved during the gavage period. Moreover, the three-day shelf life was also suitable for conducting the assays, but we did not evaluate a maximum shelf life for the CFAB.

**Fig 2:**
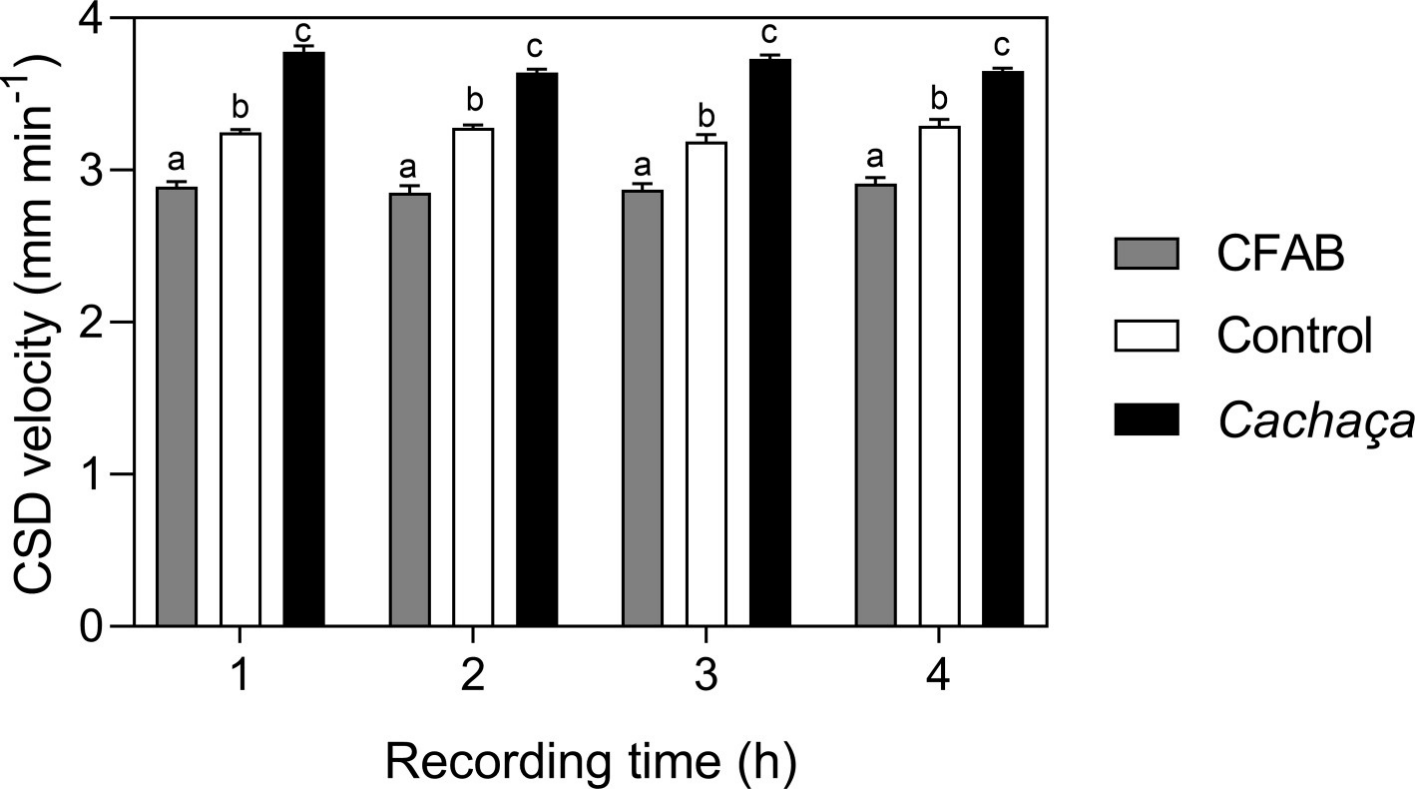
Electrophysiological recordings during cortical spreading depression effect of the alcohol consumption (CFAB, *cachaça* and only water) on cortical spreading depression propagation velocities in young-adult rats.

We observed a reduction of CSD-velocity in young-adult rats (at 79–88 days of life) that consumed CFAB, lower than the control group (water). The CSD–velocity in adult rats was significantly different in all treatments (p <0.05), and no significant difference was observed over the four-hour section for the same treatment (Fig 2).

Several studies have demonstrated the ability of substances that facilitate the spread of CSD, such as monosodium glutamate [40] and tianeptine [41], and other compounds that prevent the spread of this electrophysiological phenomenon, such as lectins [42]. In particular, ethanol has different effects depending on the concentration or exposure frequency, that can facilitate the CSD, and this justifies the higher CSD velocity in *cachaça*-group rats treated. Abadie-Guedes et al. [28] demonstrated that the antioxidant compounds exert an antagonistic action against the effect of chronic EtOH on CSD in rats. These authors found that a dose of astaxanthin alone was able to counteract EtOH action on CSD, suggesting that this effect is related to the antioxidant activities of this molecule and its protective effect on the brain. CSD is a wave of neuronal and glial depolarization, which is slowly propagated in the cortex (3–5 mm min^-1^), and followed by a long-lasting suppression of neuronal activity and excitability, also observed by Bogdanov et al. [43]. Interestingly, in the present study, CFAB-group had a lower CSD velocity than even the water-group treated, in short, this information suggests that consumption of CFAB exert an interesting effect in protecting the brain. Furthermore, for both CFAB and water the CSD-velocity remained close to the order of 3 mm min^-1^ –velocity reported for comfortable situations.

**Fig 3:**
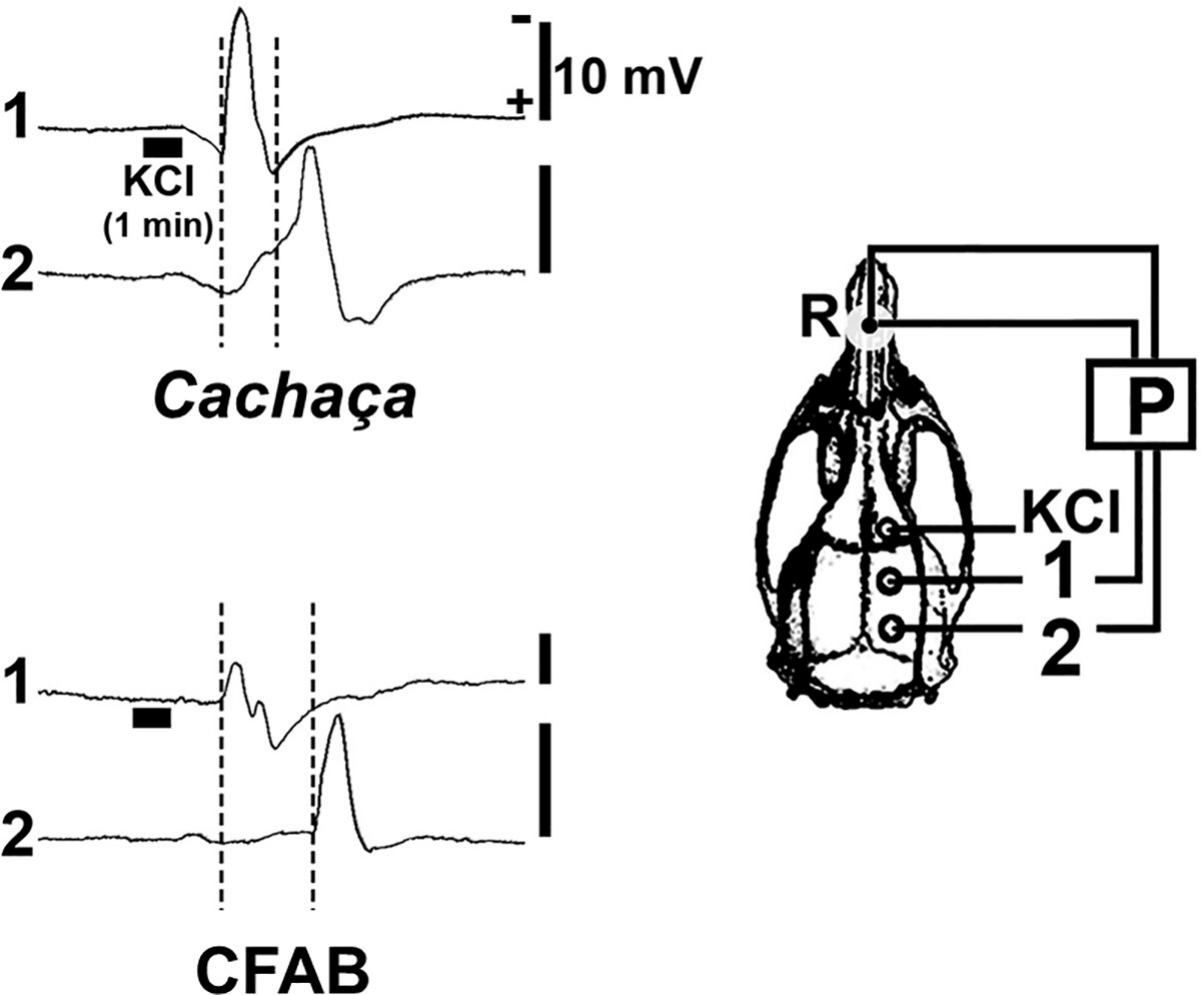
Recordings of spontaneous cortical electrical activity (ECoG; two upper traces in each panel) and slow potential change (P; two lower traces) during spreading depression in rats. Spreading depression was elicited by applying a cotton ball (1–2 mm diameter) soaked in 2% KCl solution for 1 min, on the frontal cortex. Vertical calibration bars equal 1 mV for the ECoG- and 10 mV for the P-recordings.

The electrophysiological recordings (electrocorticogram and slow cortical depression-potential changes) during CSD are show in Fig 3. Application of KCl for 1 min on a cortical point in the frontal region has been an effective technique in eliciting a single spreading depression-wave [26]. In the present study, waves have propagated without interruption and was recorded in the parietal region of the same hemisphere, as documented by the electrophysiological recordings.

In recent decades, considerable scientific efforts have been made to determine whether antioxidant supplementation can help prevent various neurological dysfunctions. The implication is that understanding the specific molecular effects of reactive oxygen species (ROS) on brain functioning is of great importance in determining the role of antioxidants on these diseases and may help to shed light on epidemiological findings, which sometimes point to contrasting neuropsychological effects of antioxidants [44]. Chronic alcoholism represents a well reported scenario of increased ROS production and, under such a condition, carotenoids can protect tissues from alcohol-induced injuries by capturing free radicals and improving the redox balance [26, 45]. In addition, other studies have demonstrated the brain tissues protection in rats by antioxidant compounds in CDS assays [27, 28, 40]. Our findings contribute to establishing a correlation between the antioxidant activities of microalgae and their neuroprotective effects in rats.

Despite this, it is necessary to assess the antioxidant power of a single molecule (or a combination of molecules) from microalgae biomass. Some “minor carotenoids” (such as neoxanthin, violaxanthin and β-carotene) also have functional properties. For example, β–carotene may play an important role in preventing degenerative diseases due to its associated antioxidant and pro-vitamin A activity [46, 47].

The data obtained showed that the rats that consumed the CFAB had a lower CSD propagation velocity. This may be associated to a protective action from *C*. *vulgaris* antioxidants compounds. ROS can be formed in the metabolism of ethanol and the presence of antioxidants may help protect tissues from the deleterious effects of ethanol. Lutein, chlorophyll-a and -b, and pheophytin a, were the most abundant antioxidants in the CFAB. These four compounds contributed to almost 50% of the total antioxidant potential in the CFAB, with their isomers and derivatives comprising the rest. This result indicates that hydrophilic compounds, lutein, chlorophylls a and b, and pheophytin a were major contributors to brain protection in rats [30]. Experimental increasing or decreasing of the brain’s ability to counteract CSD may help to understand the phenomenon and the diseases related to it.

According to Zuccalà et al. [6], studies on the potential benefits of alcohol use should not be banned from current research. In fact, the results of this study, together with those focusing on the favorable cardiovascular effects of alcohol consumption, indicate that moderate drinking may confer health benefits that should not be disregarded, from the perspectives of both the patients and society as a whole. Several research groups have been formed to investigate the neural protective effects of antioxidant molecules, mainly those from nutraceutical- and dietary sources, both in laboratory animals and in humans, using *in vivo* and *in vitro* models. Therefore, it is suggested that *C*. *vulgaris* biocompounds extracted with *cachaça* can be used as a functional alcoholic beverage that provides protection against the deleterious effects of ethanol on the brain, when moderately consumed. Nevertheless, it is worth noting that our objective was not to encourage or discourage the consumption of alcoholic beverages, but to highlight the advantages and potential for the use of microalgae biomass by the alcohol industry, contributing to the supply of functional alcoholic beverages.

## Conclusions

The findings presented in this study provide evidence that an alcoholic beverage containing the microalga *C*. *vulgaris* contains compounds with antioxidant activity and exert antioxidant effect *in vivo* and *in vitro*. This was demonstrated by resistance to CDS in the cerebral cortex of young-adult rats that consumed the beverage, which indicates a neuroprotective action of the functional beverage. The action from this functional alcoholic beverage, in this context, is greater than what might have been expected from the *cachaça* content alone. The antioxidant compounds in *C*. *vulgaris* may play a role in preventing oxidative damage to the brain by free radicals.

### Informed consent, human/animal rights

The rats utilized in this study were handled in accordance with the "Principles of Laboratory Animal Care" (National Institutes of Health, USA) and with the norms of the Ethics Committee for Animal Research of the Universidade Federal de Pernambuco.

## Supporting information

S1 Graphical abstract (TIF)

## Abbreviations

CFAB: *Chlorella vulgaris* functional alcoholic beverage
CSD: cortical spreading depression
DC: direct current
DPPH– 1: 1-diphenyl-2-picryl hydrazyl
ROS: reactive oxygen species
