# Supplementary figures and images for: Chlorella vulgaris functional alcoholic beverage: Effect on propagation of cortical spreading depression and functional properties
Source: PLoS One. 2021 Aug 9;16(8):e0255996. doi: 10.1371/journal.pone.0255996 (PMC8351948; doi:10.1371/journal.pone.0255996)

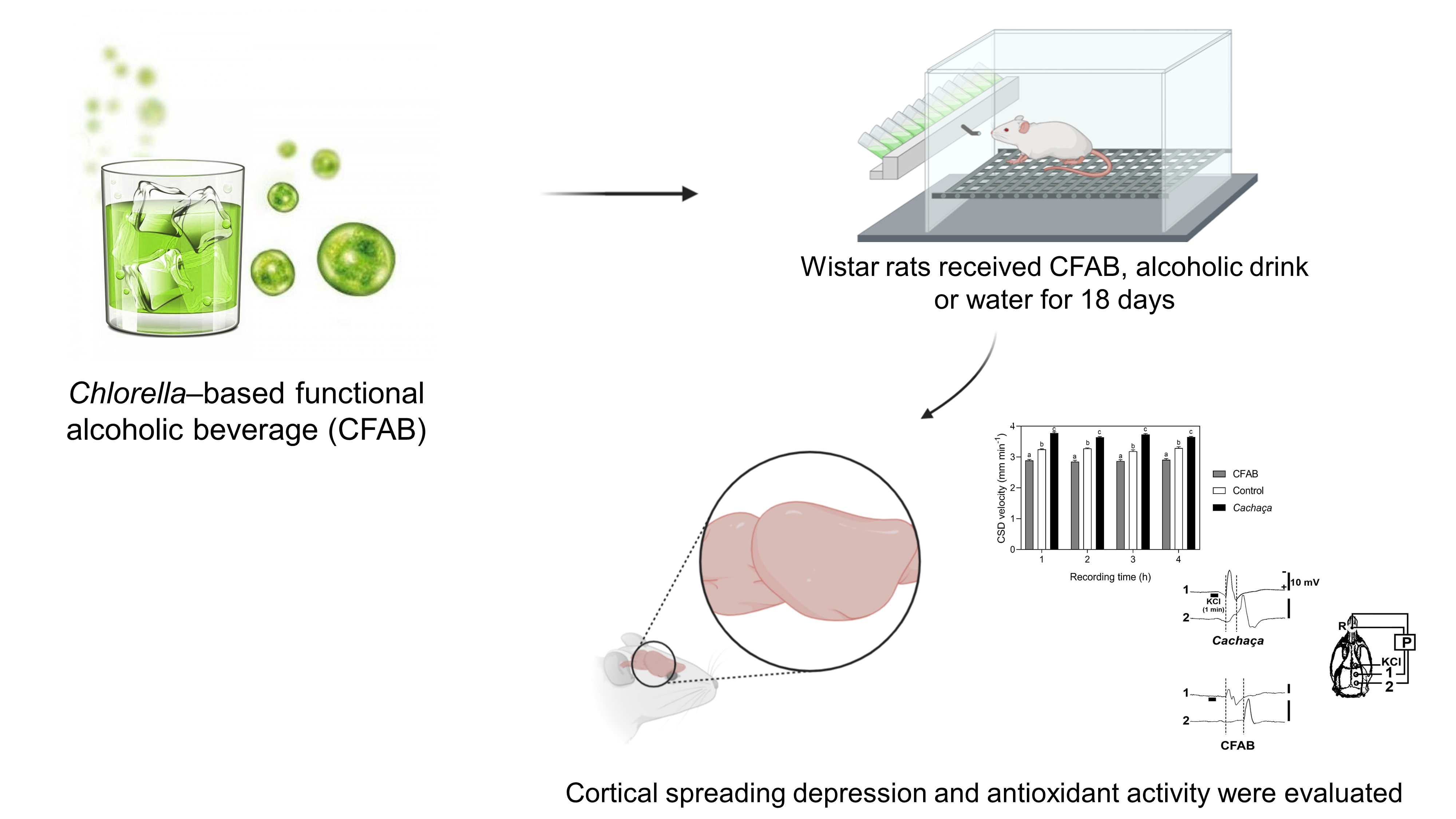

Supplement: S1 Graphical abstract — (TIF) [file pone.0255996.s001.tif]
